# Activity of the dietary flavonoid, apigenin, against multidrug-resistant tumor cells as determined by pharmacogenomics and molecular docking

**DOI:** 10.1186/2051-1426-3-S1-P10

**Published:** 2015-08-14

**Authors:** Mohamed Saeed, Thomas Efferth, Onat Kadioglu, Hassan Khalid, Yoshikazu Sugimoto

**Affiliations:** 1 Department of Pharmaceutical Biology, Institute of Pharmacy and Biochemistry - Johannes Gutenberg-University, Mainz, Germany; 2 Department of Pharmacognosy, University of Khartoum, Khartoum, Sudan; 3 Division of Chemotherapy, Faculty of Pharmacy - Keio University, Tokyo, Japan

## 

Natural products have been extensively studied and involved in cancer therapy field [1], apigenin has considerable cytotoxic activity in vitro and in vivo. Despite many mechanistic studies, less is known about resistance factors hampering apigenin’s activity. We investigated the ATP-binding cassette (ABC) transporters BCRP/ABCG2, P-glycoprotein/ABCB1 and its close relative ABCB5. Apigenin inhibited not only P-glycoprotein, but also BCRP by increasing cellular uptake of doxorubicin and showed synergistic inhibitory effect in combination with doxorubicin or docetaxel against multidrug-resistant cells. To perform in silico studies, we first generated homology models for human P-glycoprotein and ABCB5 based on the crystal structure of murine P-glycoprotein. Their nucleotide binding domains (NBDs) revealed the highest degrees of sequence homologies (89%-100%), indicating that ATP binding and cleavage is of crucial importance for ABC transporters. In silico studies showed a pigenin bound to the NBDs of P-glycoprotein and ABCB5. Hence, apigenin may compete with ATP for NBD-binding leading to energy depletion to fuel the transport of ABC transporter substrates. Furthermore, we performed COMPARE and hierarchical cluster analyses of transcriptome-wide mRNA expression profiles of the National Cancer Institute tumor cell line panel. Microarray-based mRNA expressions of genes of diverse biological functions significantly predicted responsiveness of tumor cells to apigenin [2].

## References

[B1] Newman DJ Cragg GM Natural products as sources of new drugs over the 30 years from 1981 to 2010 J Nat Prod 2012 75 3 311 35 10.1021/np200906s 22316239 PMC3721181

[B2] Saeed M Kadioglu O Khalid H Sugimoto Y Efferth T Activity of the dietary flavonoid, apigenin, against multidrug-resistant tumor cells as determined by pharmacogenomics and molecular docking J Nutr Biochem 2015 26 1 44 56 10.1016/j.jnutbio.2014.09.008 25459885

